# Impact of Ambient PM₁₀ and SO₂ Levels on Intensive Care Unit Admissions Due to Cardiopulmonary Diseases in a Tertiary Care Hospital

**DOI:** 10.34172/aim.34530

**Published:** 2025-09-01

**Authors:** Melike Yüksel Yavuz, Hüseyin Döngelli, Mehmet Yavuz, Adem Şahin, Murat Güneş, Işıl Köse Güldoğan, Nimet Şenoğlu

**Affiliations:** ^1^Katip Celebi University, Faculty of Medicine, Pulmonology Department, Izmir, Turkiye; ^2^Buca Seyfi Demirsoy Training and Research Hospital, Internal Medicine Department, Izmir, Turkiye; ^3^Tepecik Training and Research Hospital, Intensive Care Department, Izmir, Turkiye

**Keywords:** Air pollution, Intensive care unit, In-hospital mortality, Particulate matter, Sulfur dioxide

## Abstract

**Background:**

Ambient air pollution, especially particulate matter (PM₁₀) and sulfur dioxide (SO₂), has been implicated in exacerbating cardiopulmonary diseases. While emergency department visits have been widely studied, the impact of pollution on intensive care unit (ICU) admissions and mortality is less understood. This retrospective observational study aimed to evaluate the association between monthly air pollutant levels and ICU admissions for cardiopulmonary conditions, as well as in-hospital mortality.

**Methods:**

We retrospectively analyzed 6,112 ICU admissions in a tertiary hospital from January 2012 to November 2019. Using defined inclusion criteria, 227 pulmonary and 344 cardiovascular ICU admissions were selected. Monthly PM₁₀ and SO₂ levels were obtained from official air monitoring stations. A one-month lag model was applied for cardiovascular admissions. Multivariate models were used to assess associations, and results were reported with 95% confidence intervals (CIs).

**Results:**

Higher PM₁₀ levels were significantly associated with pulmonary ICU admissions (β=0.017; 95% CI: 0.003–0.031; *P*=0.020) and with cardiovascular admissions using a one-month lag structure (β=0.018; 95% CI: 0.005–0.030; *P*=0.006). SO₂ showed no significant associations. No significant relationship was observed between air pollution and in-hospital mortality. Chronic kidney disease (HR=1.309; 95% CI: 1.031–1.663; *P*=0.027) and higher Simplified Acute Physiology Score (SAPS) scores (HR=1.012; 95% CI: 1.006–1.017; *P*<0.001) were independent mortality predictors.

**Conclusion:**

This study indicates that long-term exposure to PM₁₀ significantly affects ICU hospitalization rates for both pulmonary and cardiac conditions, particularly reflecting delayed effects in cardiovascular admissions, without a corresponding impact on in-hospital mortality.

## Introduction

 Air pollution is among the most significant environmental factors with substantial short- and long-term adverse effects on human health.^1^ Particulate matter with a diameter of 10 micrometers or smaller (PM_10_) and sulfur dioxide (SO_2_) are among the most concerning air pollutants due to their pervasive occurrence and well-documented associations with negative health effects.These substances have been shown to worsen chronic illnesses, provoke acute medical events, and contribute to higher rates of hospital admissions, especially in high-risk groups such as older adults and individuals with underlying health conditions.^2^

 Multiple factors, such as pollutant concentration, length of exposure, seasonal fluctuations, and presence of comorbidities, can influence the impact of air pollution on human health.^3^ Although numerous studies have assessed how air pollution affects emergency room visits and overall hospital admissions, limited research has specifically addressed its association with admissions to intensive care units (ICUs), which often represent more critical clinical conditions.^4-6^ Extensive epidemiological research has linked both short- and long-term exposure to PM_10_ and SO_2_ with a heightened risk of developing respiratory conditions such as asthma, chronic obstructive pulmonary disease (COPD), and pneumonia.^7,8^ Likewise, exposure to air pollutants has been associated with adverse cardiovascular events, including heart failure, ischemic heart disease, and arrhythmias.^9^ While several studies have explored the association between air pollution and ICU admissions, as well as in-hospital mortality, the nature of this relationship remains insufficiently understood.^10,11^

 Moreover, the biological plausibility of pollutant-related ICU admissions is supported by several mechanistic studies. PM_10_ and SO_2_ have been shown to induce oxidative stress, systemic inflammation, endothelial dysfunction, and autonomic imbalance, all of which can precipitate acute respiratory or cardiovascular decompensation requiring ICU care.^12^ As one of the world’s megacities, Tehran, Iran, experiences significant seasonal variations in pollutant levels, particularly during the colder months, due to atmospheric inversion combined with heavy traffic and urban-industrial emissions. According to statistics from the Iranian Ministry of Health, ischemic heart disease and respiratory illnesses consistently rank among the leading causes of hospitalization and mortality.^13,14^ We hypothesized that monthly exposure to air pollutants, specifically PM_10_ and SO_2_, may influence ICU admissions due to pulmonary or cardiovascular events. This study aimed to investigate this potential association and to identify factors related to in-hospital mortality.

## Patients and Methods

###  Patients and Data Collection

 This retrospective observational single-center study was conducted using data from patients who were admitted to the ICU between January 2012 and November 2019. A total of 6,112 ICU admissions were initially reviewed, from which 227 patients with pulmonary conditions and 344 patients with cardiovascular conditions were selected for analysis. Pulmonary ICU admissions were defined based on ICD-10 codes J12–J18 (pneumonia), J44 (COPD), and J80 (ARDS). Diagnoses were confirmed by attending physicians and coded within the hospital information system. The inclusion criteria focused on adult patients (aged 18 years and older) who were diagnosed with either pulmonary or cardiac conditions and who required ICU care during the specified study period. The exclusion criteria included patients with incomplete clinical data, trauma cases, sepsis-related ICU admissions, surgical ICU cases, and individuals under 18 years of age at the time of admission. Patient clinical data, including demographic information (age and sex), comorbidities, prescribed medications, reasons for ICU admission, and routine laboratory test results, were retrieved from the hospital’s electronic health records system. The database used for data collection was maintained by the hospital’s Information management system, which ensured accurate and comprehensive data retrieval.

 The pulmonary conditions included in the analysis were primarily pneumonia, COPD, and acute respiratory distress syndrome (ARDS), while the cardiac conditions primarily consisted of congestive heart failure, acute coronary syndrome, and arrhythmias. The severity of illness at the time of ICU admission was assessed using the Simplified Acute Physiology Score II (SAPS II), a validated tool for predicting mortality and morbidity in critically ill patients. Monthly average concentrations of PM_10_ (µg/m³) and SO_2_ (ppm) were obtained from the İzmir Metropolitan Municipality Air Quality Monitoring System, specifically from the station closest to the hospital (within a 20-km radius). Data were derived from two continuous monitoring stations with > 95% data completeness. Missing daily values ( < 2%) were imputed using linear interpolation.

 To investigate temporal dynamics, lag structures were defined as lag 0 (same month), lag 1 (previous month), and lag 2 (two months prior). The final analysis focused on lag 1, based on prior literature suggesting delayed inflammatory responses to pollution exposure. This study was reported in accordance with the STROBE (Strengthening the Reporting of Observational Studies in Epidemiology) guidelines for observational research.

###  Statistical Analysis

 All analyses were conducted using the Statistical Package for the Social Sciences (SPSS) version 24.0. Normality was tested using the Kolmogorov-Smirnov and Shapiro-Wilk tests. Parametric continuous variables were compared with Student’s T test, while the Mann-Whitney U test was used for non-parametric continuous variables. Results were presented as mean ± standard deviation (SD) and median (interquartile range). Categorical variables were compared using the Chi-Square and Fisher’s exact tests, with results reported as n (%). This study aimed to investigate the relationship between air pollution levels (PM_10_ and SO_2_) and monthly ICU admission rates using a generalized linear model. Data on air pollution levels and ICU admissions were collected over an eight-year period (2012–2019). Monthly averages of PM_10_ (µg/m³) and SO_2_ (ppm) were used for the independent variables, while the total number of ICU admissions per month was the dependent variable. To examine the delayed effects of air pollution, lagged values of PM_10_ and SO_2_ from the previous month were included in the model. Pulmonary and cardiac ICU hospitalization rates were analyzed as separate dependent variables. In-hospital mortality was assessed using Cox regression and Kaplan-Meier analyses. The relationship between air pollution (PM_10_ and SO_2_) and monthly ICU admissions was assessed using a generalized linear regression model with a log-link function, assuming a Poisson distribution, appropriate for count data. Model diagnostics, including deviance, overdispersion, and residual analysis, were evaluated. Covariates adjusted for included age, sex, and seasonality (warm vs. cold months). Statistical significance was determined using p-values, with a threshold of 0.05. Confidence intervals (CIs) were reported at the 95% level for all effect estimates.

## Results

###  Clinical Characteristics of Study Group

 This study involved 571 patients with a median age of 70 years, 60% of whom were female. During the study period, all patients ICU admissions were recorded, of which 227 (40%) were due to pulmonary conditions and 344 (60%) due to cardiac conditions. The median duration of ICU hospitalization was 8 days. The most frequent diagnoses were pneumonia (32.6%), congestive heart failure (28.9%), and acute coronary syndrome (15.9%). Cardiovascular disease (33.3%) and diabetes mellitus (27.7%) were the most common comorbidities. The ICU mortality rate was 41.1%, while in-hospital mortality was 36.2% (Table 1).

**Table 1:** Demographic and Clinical Characteristics of Patients

	**Total (n=571)**
Age median (IQR)	70 (60‒79)
Gender n (%)	
Female	342 (60)
Male	229 (40)
Hospitalization duration days median (IQR)	8 (3‒23)
SAPS II score median (IQR)	59 (47.25‒72)
Laboratory findings median (IQR)	
Creatinine	1.4 (1.1‒2.5)
Albumin	2.9 (2.5‒3.4)
WBC	14.9 (11.1‒19.9)
CRP	76.1 (25.3‒165.7)
Procalcitonin	1.41 (0.36‒7.75)
Diagnosis *n* (%)	
Pneumonia	186 (32.6)
COPD exacerbation	51 (8.9)
Pulmonary embolism	8 (1.4)
Congestive heart failure	165 (28.9)
Acute coronary syndrome	91 (15.9)
Other causes	70 (12.3)
Comorbidities *n* (%)	
Cardiovascular disease	190 (33.3)
COPD	98 (17.2)
Diabetes mellitus	158 (27.7)
Chronic kidney disease	89 (15.5)
Cerebrovascular accident	37 (6.5)
Malignancy	34 (6)
Admission origin *n* (%)	
Emergency room	168 (29.4)
Wards	254 (44.5)
Other	149 (26.1)
Mortality *n *(%)	
Intensive care unit	235 (41.1)
In-hospital	207 (36.2)

COPD, Chronic obstructive pulmonary disease; CRP, C-reactive protein; IQR, Interquartile range; SAPS II, Simplified acute physiology score; WBC, White blood cell.

###  Comparison of Pulmonary and Cardiac Admissions

**Figure 1:**
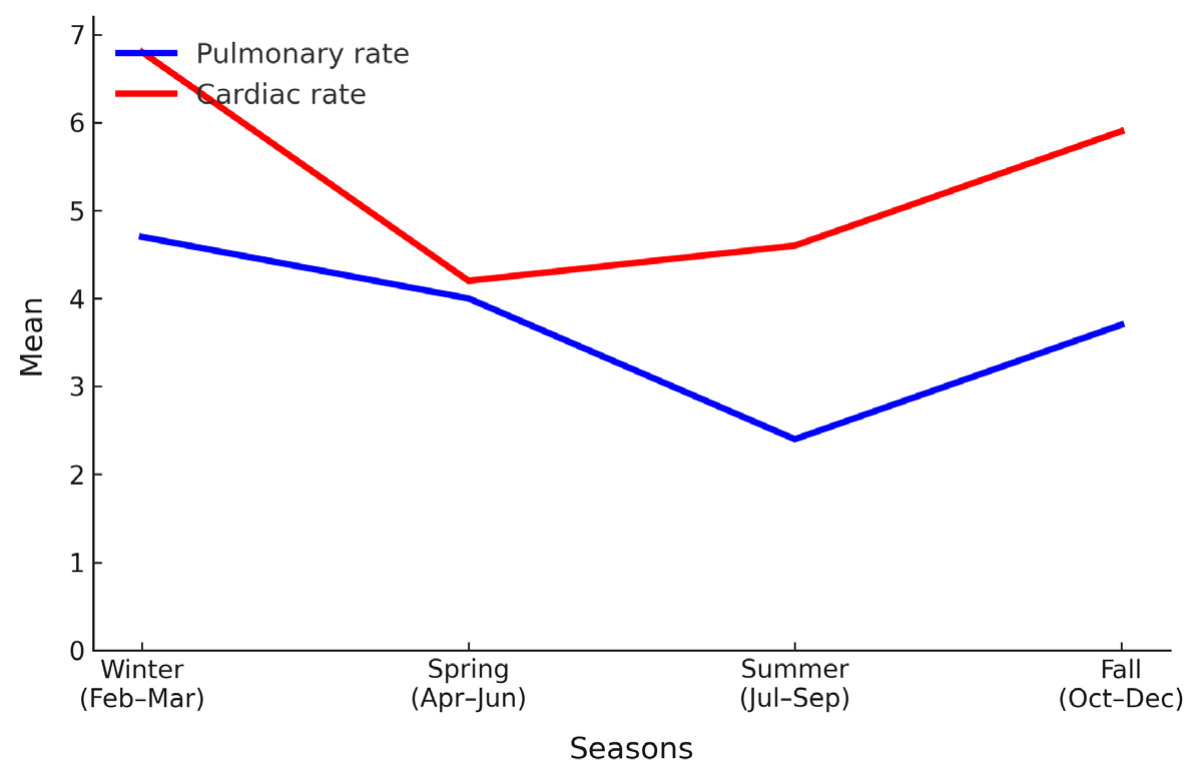
Rate of Hospitalized Patients in the ICU Due to Pulmonary and Cardiac Causes Across Multiple Sessions.

 Pulmonary patients (n = 227) had a median age of 69, while cardiac patients (n = 344) had a median age of 70 (*P* = 0.922), with no significant gender differences (*P* = 0.596). Pulmonary patients had longer ICU hospitalization (11 days vs. 7 days, *P* = 0.009) and higher SAPS II scores (60.2 vs. 60, *P* = 0.003). Significant differences were found in albumin (*P* < 0.001) and CRP levels (*P* < 0.001), with pulmonary patients having lower albumin and higher CRP. Pulmonary patients had higher rates of COPD (28.2% vs. 9.9%, *P* < 0.001) and chronic kidney disease (CKD) (52.9% vs. 49.1%, *P* = 0.382), while cardiac patients had more cardiovascular disease (50.6% vs. 7%, *P* < 0.001). Mortality rates were significantly higher in cardiac patients (88.1% vs. 75.3%, *P* < 0.001). SO_2_ levels were comparable between pulmonary and cardiac patient groups (median 9 ppm, IQR: 4), and the difference was not statistically significant (*P* = 0.982). This suggests that SO_2_ exposure did not differ between groups and may not have had a differential impact based on disease type. Similarly, PM_10_ levels were slightly higher in the pulmonary group compared to the cardiac group (median 42 µg/m³ vs. 39 µg/m³), but this difference was also not statistically significant (*P* = 0.280). While mean exposure levels did not vary significantly between groups, the significant associations observed in regression analyses suggest that the health effects of PM_10_ may be more closely related to temporal variability and lag effects rather than average concentrations alone (Table 2). The rate of patients hospitalized in intensive care for pulmonary and cardiac causes over multiple sessions is shown in Figure 1.

**Table 2:** Demographic and Clinical Characteristics of Patients

	**Pulmonary (** ***n*** **=227)**	**Cardiac (** ***n*** **=344)**	***P*** ** value**
Age median (IQR)	69 (22)	70 (19)	0.922
Gender n (%)			0.596
Female	88 (38.8)	141 (41)	
Male	139 (61.2)	203 (59)	
Hospitalization duration days median (IQR)	11 (22)	7 (17)	0.009
SAPS II score median (IQR)	60.2 (14)	60 (13)	0.003
Laboratory findings median (IQR)			
Creatinine mg/dL	1.6 (1.7)	1.4 (1.2)	0.885
Albumin g/dL	2.7 (0.8)	3.1 (0.9)	< 0.001
WBC 10*3	13.9 (9)	15.5 (9)	0.075
CRP mg/dL	96.7 (57.2)	67 (120)	< 0.001
Procalcitonin IU/dL	1.17 (6.23)	1.71 (7.09)	0.212
Comorbidities n (%)			
Cardiovascular disease	16 (7)	174 (50.6)	< 0.001
COPD	64 (28.2)	34 (9.9)	< 0.001
Diabetes mellitus	86 (37.9)	73 (21.2)	< 0.001
Chronic kidney disease	120 (52.9)	169 (49.1)	0.382
Cerebrovascular accident	23 (10.1)	14 (4.1)	0.004
Malignancy	28 (12.3)	6 (1.7)	< 0.001
PM_10_ µg/m³median (IQR)	42 (18)	39 (14)	0.280
SO_2_ ppmmedian (IQR)	9 (4)	9 (4)	0.982
Mortality n(%)	171 (75.3)	303 (88.1)	< 0.001

COPD, Chronic obstructive pulmonary disease; CRP, C-reactive protein; IQR, Interquartile range; SAPS II, Simplified acute physiology score; WBC, White blood cell; PM_10_, Particulate matter 10 micrometers or less in diameter; SO_2_, Sulfur dioxide.

###  Factors Associated with Pulmonary ICU Hospitalization Rate in Generalized Linear Models

**Figure 2:**
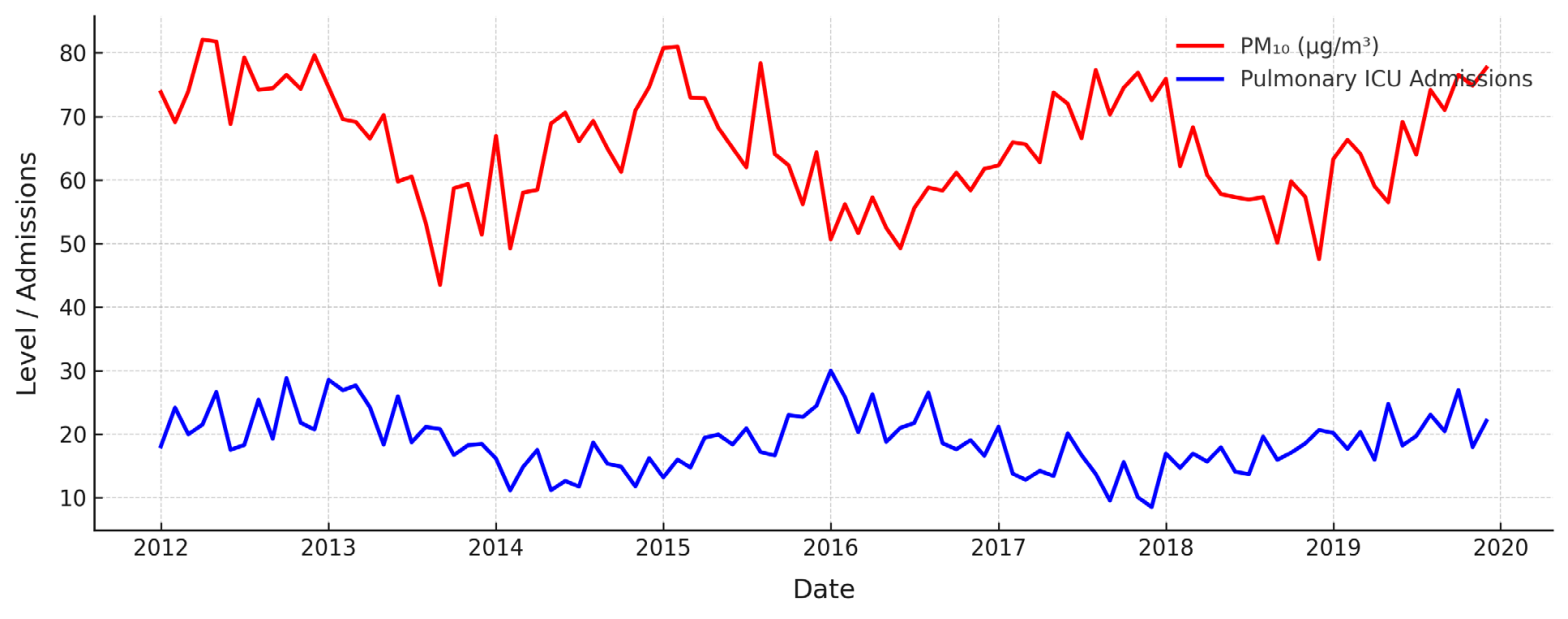
Monthly PM₁₀ Levels and Pulmonary ICU Admissions

 Univariate analysis revealed that PM_10_ exposure was significantly associated with an increase in pulmonary ICU hospitalization rates (β = 0.014, *P* = 0.015), while age, SO_2_, and seasonal variations showed no significant effects (*P* > 0.05). In multivariate analysis, PM_10_ remained a significant factor (β = 0.017, *P* = 0.020), indicating a positive correlation with pulmonary ICU hospitalization rates. Additionally, a Spearman correlation analysis was performed to further assess the relationship between PM_10_ levels and pulmonary ICU admissions. The analysis revealed a statistically significant moderate positive correlation (r = 0.76, *P* = 0.004), reinforcing the association identified in the generalized linear regression model. Figure 2 illustrates the temporal association between monthly average PM_10_ levels (µg/m³) and the number of pulmonary ICU admissions over eight years. A parallel upward trend in PM_10_ concentration and pulmonary ICU admissions was observed, particularly during winter months, suggesting a potential seasonal pattern. However, age, SO_2_, and seasonal variations (winter, spring, summer) did not demonstrate significant associations in either the univariate or multivariate models (*P* > 0.05) (Table 3).

**Table 3:** Generalized Linear Model Analysis of Pulmonary ICU Hospitalization Rate with Related Factors

	**Univariate Analysis**			**Multivariate Analysis**		
	**β**	**95% CI**	***P*** ** value**	**β**	**95% CI**	***P*** ** value**
Age	0.008	-0.014/0.029	0.480	0.026	-0.015/0.026	0.538
PM_10_ (µg/m³)	0.014	0.003/0.026	0.015	0.017	0.003/0.032	0.020
SO_2_ (ppm)	0.008	-0.036/0.051	0.725	-0.009	-0.058/0.039	0.704
PM_10_ Lag1	0.011	-0.001/0.022	0.065			
SO_2_ (ppm)Lag1	0.007	-0.033/0.048	0.724			
Session (Winter)	0.190	-0.154/0.535	0.279	0.189	-0.145/0.523	0.268
Session (Spring)	0.087	-0.257/432	0.619	0.322	-0.108/0.752	0.142
Session (Summer)	-0.300	-0.659/0.059	0.101	-0.076	-0.503/0.350	0.725
Session (Fall)	Reference					

CI, Confidence interval; OR, Odds ratio; PM_10_, Particulate matter 10 micrometers or less in diameter; SO_2_, Sulfur dioxide; β, Beta coefficient.

###  Factors Associated with Cardiac ICU Hospitalization Rate in Generalized Linear Models

 In the univariate analysis, age, SO_2_, or seasonal variations did not show any significant associations with cardiac ICU hospitalization rates (*P* > 0.05). However, PM_10_ exposure showed a marginal association (β = 0.008, *P* = 0.199). In the multivariate analysis, only PM_10_ Lag1 (previous month’s exposure) demonstrated a significant positive relationship with cardiac ICU hospitalization rates (β = 0.018, *P* = 0.006). Age, SO_2_, and seasonal variations did not show significant associations in either analysis (*P* > 0.05) (Table 4).

**Table 4:** Generalized linear model analysis of cardiac ICU hospitalization rate with related factors

	**Univariate Analysis**			**Multivariate Analysis**		
	**β**	**95% CI**	***P*** ** value**	**β**	**95% CI**	***P*** ** value**
Age	-0.001	-0.020/0.019	0.939	-0.006	-0.026/0.013	0.519
PM_10_ (µg/m³)	0.008	-0.004/0.020	0.199			
SO_2_ (ppm)	0.005	-0.036/0.047	0.804	-0.022	-0.066/0.023	0.337
PM_10_ (µg/m³) Lag1	0.015	0.003-0.027	0.011	0.018	0.005/0.031	0.006
SO_2_ (ppm)Lag1	0.014	-0.029/0.057	0.517			
Session (Winter)	0.184	-0.159/0.529	0.292			
Session (Spring)	-0.217	-0.568/0.134	0.226			
Session (Summer)	-0.144	-0.490/0.203	0.416			
Session (Fall)	Reference					

CI, Confidence interval; OR, Odds ratio; PM_10_, Particulate matter 10 micrometers or less in diameter; SO2, Sulfur dioxide; β, Beta coefficient.

###  Factors Associated with In-hospital Mortality

 In the univariate analysis, age, SAPS score, cardiac diagnosis, infection, CKD, and diabetes mellitus (DM) were significantly associated with survival (*P* < 0.05). Specifically, older age (HR = 1.009, *P* = 0.008) and higher SAPS score (HR = 1.016, *P*< 0.001) were strong predictors of poor survival. Cardiac diagnosis also increased the risk (HR = 1.316, *P* = 0.004). Interestingly, infection was associated with a reduced risk of in-hospital mortality in our cohort (HR = 0.768, *P* = 0.004). This seemingly paradoxical result may be due to selection bias or differential ICU admission thresholds, where patients admitted with isolated infections (particularly pneumonia) may have had fewer comorbidities or better physiological reserves compared to those admitted with decompensated cardiac conditions or multi-organ failure. Additionally, infections may have prompted earlier ICU hospitalization and intervention, contributing to improved outcomes relative to other critically ill patients. In the multivariate analysis, only SAPS score (HR = 1.012, *P* < 0.001) and CKD (HR = 1.309, *P* = 0.027) remained significant predictors of survival, while the impact of age and cardiac diagnosis became non-significant. The effects of environmental factors like PM_10_ and SO_2_ exposure were not significant in either analysis (Table 5).

**Table 5:** Cox Regression Analysis of Survival with Related Factors

	**Univariate analysis**			**Multivariate analysis**		
	**HR**	**95% CI**	***P*** ** value**	**HR**	**95% CI**	***P*** ** value**
Age	1.009	1.002‒1.015	0.008	1.006	0.999‒1.014	0.116
Female gender	1.005	0.836‒1.208	0.961	1.027	0.846‒1.246	0.789
SAPS score	1.016	1.010‒1.021	< 0.001	1.012	1.006‒1.018	< 0.001
Cardiac versus pulmonary	1.316	1.091‒1.588	0.004	1.143	0.850‒1.538	0.375
PM_10_	0.995	0.987‒1.002	0.157	0.995	0.986‒1.004	0.295
SO_2_ (ppm)	0.974	0.944‒1.005	0.105	0.993	0.956‒1.030	0.694
PM_10_ (µg/m³) Lag1	0.996	0.988‒1.003	0.269			
SO_2_ (ppm)Lag1	0.975	0.946‒1.005	0.096			
Infection	0.768	0.641‒920	0.004	0.834	0.647‒1.075	0.161
CVD	1.144	0.946‒1.384	0.167	1.024	0.814‒1.289	0.839
COPD	0.854	0.661‒1.104	0.228	0.951	0.724‒1.249	0.719
CKD	1.506	1.255‒1.807	< 0.001	1.309	1.031‒1.662	0.027
CVA	0.693	0.463‒1.037	0.074	0.927	0.612‒1.403	0.720
DM	1.336	1.098‒1.627	0.004	1.142	0.890‒1.465	0.298
Malignancy	1.202	0.825‒1.752	0.336			
Procalcitonin	1.001	0.997‒1.003	0.981			
CRP	1.001	0.999‒1.001	0.663			
WBC	1.006	0.996‒1.017	0.249	1.009	0.998‒1.020	0.099
Creatinine	1.085	1.035–1.139	0.001	1.020	0.946‒1.100	0.600
Albumin	0.967	0.871‒1.072	0.522			

CI, Confidence interval; HR, hazard ratio; SAPS, Simplified acute physiology score; PM_10_, Particulate matter 10 micrometers or less in diameter; SO_2_, Sulfur dioxide; CVD, Cardiovascular disease; COPD, Chronic obstructive pulmonary disease; CKD, Chronic kidney disease; CVA, Cerebrovascular accident; DM, Diabetes mellitus; CRP, C-reactive protein; WBC, White blood cells.

## Discussion

 Our findings demonstrated that long-term exposure to PM_10_ was significantly associated with an increased rate of pulmonary ICU admissions, both in univariate and multivariate models. Although the observed differences in PM_10_ exposure and ICU admission rates were statistically significant, the effect sizes also suggest clinical relevance, particularly in the context of public health policy and resource allocation for respiratory care. Similarly, lagged PM_10_ exposure (from the previous month) showed a significant relationship with cardiac ICU admissions, suggesting a potential delayed effect of air pollution on cardiovascular morbidity. In contrast, no significant associations were observed between SO_2_ levels and ICU admission rates in either diagnostic group. The differential associations observed (PM_10_ with both cardiac and pulmonary ICU admissions, and SO_2_ with no significant outcomes) may reflect the broader systemic effects of particulate matter compared to the more localized respiratory irritation caused by sulfur dioxide. Higher SAPS II scores and the presence of CKD emerged as significant predictors of in-hospital mortality, while air pollution parameters such as PM_10_ and SO_2_ were not independently associated with survival outcomes.

 Previous studies have typically examined short-term exposure to air pollution, often focusing on durations measured in days. These studies have similarly reported that indicators such as SO_2_ and PM_10_ are associated with increased rates of hospital admissions and higher rates of hospitalization due to pulmonary or cardiac causes.^15-18^ In our study, we aimed to explore the effects of more prolonged exposure, utilizing monthly average values of air pollutants. We observed that monthly PM_10_ concentrations were significantly associated with increased pulmonary-related admissions, while lagged PM_10_ values (reflecting exposure from the previous month) were significantly associated with cardiac-related hospitalizations. These findings suggest that the adverse health effects of air pollution persist even with extended exposure durations. In contrast to PM_10_, SO_2_ levels were not found to have a significant impact on admission rates in our study. We believe the discrepancy in findings may be due to the fact that previous studies primarily investigated the short-term effects of SO_2_, whereas our study focused on its potential long-term impact on ICU admissions.^9,18^

 Consistent with previous studies demonstrating that long-term exposure to air pollutants such as PM_10_ increases cardiovascular mortality, our findings also showed that lagged PM_10_ values were associated with higher rates of cardiovascular admissions.^19^ This suggests that, unlike the more immediate impact of air pollution on the respiratory system, its adverse effects on the cardiovascular system may manifest with a delayed response.

 Numerous studies have demonstrated that exposure to smaller particulate pollutants, such as PM_2.5_, can exacerbate both cardiac and pulmonary conditions even over very short periods, and is also associated with increased in-hospital mortality and morbidity, including prolonged duration of mechanical ventilation or hospitalization.^11,16,17,20^ However, due to the design of our study, we were unable to assess the effects of PM_2.5_. Interestingly, in an *in-vitro* lung cell study investigating genetic alterations caused by chronic exposure to PM_2.5_ and PM_10_, PM_10_ was found to induce more extensive genetic changes.^21^ In line with this, our findings showing that both prolonged and lagged PM_10_ exposure were associated with adverse outcomes may suggest that PM_10_ could potentially exert more harmful effects than PM_2.5_ in the context of chronic exposure. Our findings are also in line with the results of the large-scale cohort study conducted by Gutman et al in 2022, which included over two million adults across a major region in France. In their study, long-term exposure to ambient PM_10_, NO_2_, and O_3_ was significantly associated with both the incidence and in-hospital mortality of ARDS. Specifically, each 10 µg/m³ increase in PM_10_ exposure was associated with a 13% higher incidence of ARDS (HR: 1.13, 95% CI: 1.07–1.19) and a 15% increase in ARDS-related hospital mortality (HR: 1.15, 95% CI: 1.04–1.27). Similarly, in our study, elevated PM_10_ levels were significantly associated with increased pulmonary ICU admission rates (β = 0.017, *P* = 0.020), and this association was further supported by a moderate-to-strong positive Spearman correlation (r = 0.76, *P* = 0.004). When considered together, these findings highlight that exposure to particulate matter such as PM_10_ not only contributes to the burden of respiratory disease but may also drive more severe clinical outcomes, including the need for intensive care and increased mortality.^22^

 In our study, neither PM_10_ nor SO_2_ showed a significant association with in-hospital mortality. However, it is important to note that the pollution data used represented average values from the month preceding ICU hospital admission, and thus did not reflect real-time in-hospital exposure. While previous studies have reported a relationship between pre-admission air pollution exposure and increased mortality, our study differed in that it utilized monthly average concentrations and focused exclusively on PM_10_ and SO_2_.^11,23^ These methodological differences may explain the discrepancy in findings. Notably, among all evaluated variables, only the presence of CKD emerged as an independent predictor of in-hospital mortality. Given that the kidneys play a central role in maintaining fluid, electrolyte, and acid-base homeostasis, the presence of chronic renal dysfunction may have significantly contributed to poor outcomes in this cohort of patients admitted to the ICU for pulmonary and cardiac causes.^24^

 This study has several limitations that should be acknowledged. It was conducted in a single center, which may limit the generalizability of the findings to broader populations or different geographic regions. Exposure assessment was based on fixed-site monitoring stations rather than individual-level exposure, which may not accurately reflect each patient’s true pollutant exposure due to variations in residential proximity, indoor air quality, or personal behaviors. The retrospective design may be prone to information bias and residual confounding due to unmeasured variables. Although the completeness of environmental data was over 95%, linear interpolation was applied for < 2% of missing daily values, which may still introduce minor biases.Air pollution exposure was assessed using monthly average concentrations, which may not accurately reflect short-term exposure peaks that could trigger acute clinical events. Moreover, the analysis was limited to PM_10_ and SO_2_, as PM_2.5_ data were unavailable during the earlier years of the study period, precluding assessment of finer particulate matter known to have significant health effects. In addition, the use of fixed monitoring station data assumes uniform exposure for all individuals, without accounting for personal behaviors, time-activity patterns, or residential proximity to pollution sources, potentially resulting in exposure misclassification. The study also did not include other relevant environmental variables such as temperature, humidity, or concurrent respiratory infections like influenza, which may have acted as confounding factors. Furthermore, indoor air pollution sources and occupational exposures, both of which can influence respiratory and cardiovascular outcomes, were not evaluated. Although comorbidities were recorded, the severity of underlying conditions (e.g. stages of CKD or heart failure) and the use of medications that could modulate clinical outcomes were not incorporated into the analysis. Lastly, while the study focused on ICU admission rates and in-hospital mortality, it did not assess other clinically relevant outcomes such as duration of ICU stay, mechanical ventilation requirement, or post-discharge prognosis.

## Conclusion

 In conclusion, our findings indicate that monthly average PM_10_ concentrations are significantly associated with increased ICU admission rates, particularly for pulmonary causes. Moreover, lagged PM_10_ exposure was found to have a notable effect on cardiac ICU hospitalization rates, suggesting a delayed impact of air pollution on cardiovascular health. However, neither PM_10_ nor SO_2_ had a significant effect on in-hospital mortality, and SO_2_ levels did not show an association with ICU hospitalization rates.
